# The Present and Future of Artificial Intelligence in Urological Cancer

**DOI:** 10.3390/jcm12154995

**Published:** 2023-07-29

**Authors:** Xun Liu, Jianxi Shi, Zhaopeng Li, Yue Huang, Zhihong Zhang, Changwen Zhang

**Affiliations:** Tianjin Institute of Urology, The Second Hospital of Tianjin Medical University, Tianjin 300211, China; tmuliuxun@163.com (X.L.);

**Keywords:** artificial intelligence, urological cancer, pathology, imaging, treatment

## Abstract

Artificial intelligence has drawn more and more attention for both research and application in the field of medicine. It has considerable potential for urological cancer detection, therapy, and prognosis prediction due to its ability to choose features in data to complete a particular task autonomously. Although the clinical application of AI is still immature and faces drawbacks such as insufficient data and a lack of prospective clinical trials, AI will play an essential role in individualization and the whole management of cancers as research progresses. In this review, we summarize the applications and studies of AI in major urological cancers, including tumor diagnosis, treatment, and prognosis prediction. Moreover, we discuss the current challenges and future applications of AI.

## 1. Introduction

The term “artificial intelligence” (AI) refers to a wide range of technologies attempting to mimic human thought processes with minimal human intervention. Robotics, speech recognition, image recognition, natural language processing, and expert systems are all areas of research in this field [1]. Due to its robust, dynamic, self-learning computational power, AI is increasingly being used and researched in the medical field, including disease diagnosis, treatment plan development, and prognosis prediction [2]. With the rapid increase in computing power, the spread of electronic case systems, and the emergence of concepts such as imaging histology and pathological histology, AI is expected to revolutionize modern medicine.

**Figure 1:**
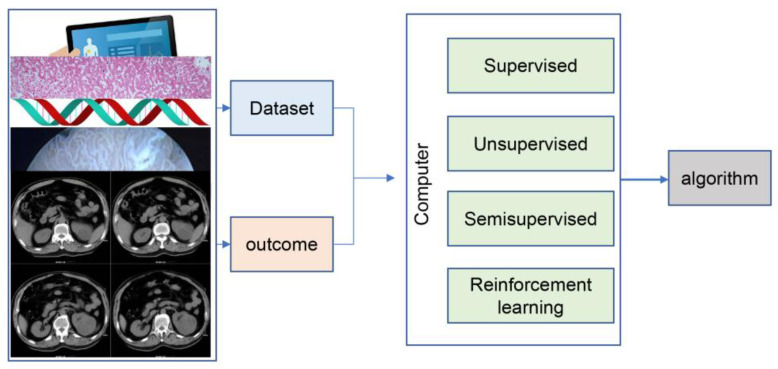
The framework of machine learning.

The number of urological cancers such as prostate, bladder, and kidney cancer is increasing every year. Prostate cancer and bladder cancer are the 4th and 10th most common cancers worldwide, respectively, and prostate cancer has become the second leading cause of cancer death in men after lung cancer [3]. With the increasing number of oncology patients and new technologies in the treatment process, doctors are facing a significant load on several elements of cancer management, including pathology, imaging, and treatment. The development of AI to assist clinicians in cancer management has been recognized as an important area of urological medical research. One of the most widely used techniques in the medical field is machine learning (ML). ML is a branch of AI that uses algorithms to automatically identify patterns and trends within the data and select features based on the task [4] (Figure 1). Deep learning (DL) is a subgroup of ML with a neural network architecture that allows models to scale exponentially with the number and dimensionality of data, enabling DL to show outstanding advantages in large-scale data processing, such as image classification and language processing [5]. Numerous published studies have demonstrated that AI has good potential for application in urological cancers, including image-guided diagnostic systems, pathology identification systems, survival prediction models, and tumor progression prediction [6]. A series of relevant prospective clinical trials have been published, showing that AI is not inferior to urologists in clinical performance. 

This review summarizes the current status and relevant research on the application of AI in the pathology, imaging, clinical treatment, and prognosis of urological cancers. Moreover, challenges and future directions that target urological cancers are also discussed to improve the applicability of AI in clinical practice.

## 2. Search Strategy and Article Selection

We systematically searched the PubMed, Cochran, and Web of Science databases for literature mainly from 2000 to 2023 for terms such as “artificial intelligence”, “deep learning”, “machine learning”, “bladder cancer”, “prostate cancer”, “kidney cancer” and “urological cancers”. Information about the first author, country of origin, study period, study design, and total number of patients was collected according to title and abstract. Then, we filtered the articles according to the following criteria.

Inclusion criteria:

1. Full-length original articles covering diagnosis, treatment planning, and prognosis related to the application of AI in urological cancer;
2. Articles with a high impact factor or high citation;
3. Articles whose authors are highly influential in the field.

Exclusion criteria:

1. Studies that were similar, duplicate, or not very relevant. The most recently published or influential articles were selected from duplicate or similar studies;
2. Literature not published in English;
3. Abstracts, review articles, and book chapters;
4. Animal, laboratory, or post-mortem studies.

**Figure 2:**
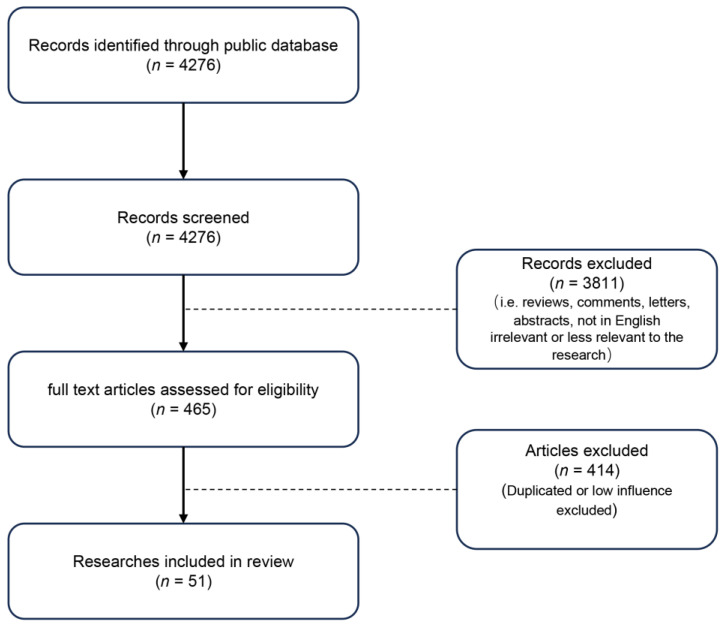
The flowchart for literature screening.

The screening process is shown in Figure 2. After reviewing the type of literature and course content, 51 studies were included in this review. 

## 3. Application of AI in Pathology

The correct histological grade or score is significant for guiding tumor treatment and evaluating patient prognosis. However, the load of pathological examinations is growing for pathologists as the prevalence of urological cancers rises. Moreover, the visual score of the pathologist is affected by subjective judgment, and the accuracy of the results is related to the pathologist’s ability. With the advancement of digital pathology, AI, especially the DL algorithm, has been widely used in pathological diagnosis [7]. AI assists pathologists in identifying benign and malignant tumors faster and more accurately, even with the correct grading and scoring. Meanwhile, AI can reduce the workload of pathologists and improve the diagnostic consistency between pathologists [8].

### 3.1. Application of AI in Kidney Cancer Pathology

Even for seasoned pathologists, it is still difficult and time-consuming to differentiate clear cell renal cell carcinoma (CCRCC) from the slice due to the complex histological features of kidney tumors. Zheng et al. used ML to identify pathological sections of kidney cancer patients and create a diagnostic and prognostic model. The diagnostic model could distinguish cancer tissue from normal tissue and CCRCC from other types of kidney cancer. Regarding survival prediction for ccRCC patients, the prediction model improved survival prediction accuracy by 6.6% for patients in Shanghai General Hospital and 2.5% for patients in the TCGA cohort compared to the current tumor staging/grading system [9]. Furthermore, Saeed et al. also developed a CNN-based AI for classifying renal cell carcinoma (RCC) based on sections. The model can precisely type surgically resected or biopsy slides into five categories. The mean accuracy of the model can be 0.97, mean precision 0.94, and mean AUC 0.98 (95% CI: 0.97–1.00) [10]. In addition, another study by Wessels et al. also constructed a prognostic prediction model by extracting features of pathological sections through convolutional neural networks (CNN). The sensitivity and specificity of the model were 72.4% and 71.7%, respectively [11]. Although the accuracy is not ideal, it opens up new ideas for prognostic prediction. 

Studies and model construction suggest that AI can significantly identify pathological sections of kidney tumors.

### 3.2. Application of AI in Bladder Cancer Pathology

Bladder urothelial cancer can be divided into low-grade and high-grade according to the degree of tumor tissue differentiation, which is crucial for individual patients with non-muscle invasive bladder cancer (NMIBC) and muscle-invasive bladder cancer (MIBC). Due to the inherent subjectivity of eye observation, the pathologist’s grading procedure exhibits rather substantial interobserver variability [12].

Using 232 specimens identified by three pathologists, Jansen et al. trained the DL model to identify and grade bladder urothelial carcinomas. The study showed that the CNN-based AI could automatically identify and appropriately grade 76% of low-grade and 71% of high-grade tumors. Although AI models are not as accurate or sensitive as pathologists, this study demonstrates the feasibility of AI for grading and identifying uroepithelial carcinoma and improves the consistency of the interpretation of results [13]. In a related study, Pan et al. developed the AI model PAIDM, which was trained on 854 whole slide images of urothelial cancer to recognize bladder tumor tissue. Then, to further increase the power of the study, they contrasted the accuracy of PAIDM and the pathologist. The result revealed that the diagnostic time of PAIDM was much faster than that of pathologists. Moreover, the AUC of accuracy was 0.847 (95% CI 0.779–0.905), which was not inferior to the average diagnostic level of pathologists [14].

### 3.3. Application of AI in Prostate Cancer Pathology

The diagnosis and treatment of prostate cancer depend on the interpretation of a prostate biopsy. The Gleason score plays a crucial role in the choice of treatment and prognosis of patients with prostate cancer. Moreover, the different interpretations of biopsy may lead to over- or under-treatment [15]. A study by Ström et al. attempted to use AI to identify puncture tumor tissue, which was trained on 6682 slices from needle core biopsies and then validated on an independent test set (271 slices) and an external validation set (1631 slices). The results showed that the AUCs of AI for prostate cancer detection and Gleason score in the external validation set were 0.997 (0.994–0.999) and 0.986 (0.972–0.996), which were consistent with the accuracy from expert pathologists [16]. Another result of the AI detection platform developed by Huang et al. after interpreting pathology slides of 589 prostate cancer patients showed that the AI system detected prostate cancer at the slice pixel level (weighted κ = 1.0; asymptotic 97% CI, 95.0–96.0) and the slide level (weighted κ = 98.0; asymptotic 98% CI, 95.0–96.1) with pathologists almost precisely, and even outperformed pathologist detection in terms of prostate cancer scoring and quantification [17]. While there is strong evidence that AI can perform the Gleason score in the same way as experienced pathologists, more work must be done to ensure the accuracy of AI systems’ results in different patient populations, digital platforms, and different settings in the pathology laboratory.

### 3.4. Application of AI in Other Cancer Pathology

Meanwhile, the incidence of other urological tumors, such as testicular cancer, upper tract urothelial carcinoma, and penile cancer, is relatively low, resulting in little research being published. Testicular germ cell tumors with a lymphovascular invasion have been demonstrated to be prognostic and challenging for pathologists to diagnose visually. Ghosh et al. attempted to locate sites of lymphovascular infiltration by using DL trained with 184 testicular cancer slide images. The final findings revealed an accuracy of 0.68, which is subpar but doable [18]. Another study conducted by Linder et al. used the DL method to recognize tumor-associated lymphocytes in the lesional tissue of 259 testicular cancers. The final model showed comparable accuracy to that of pathologists [19].

## 4. Application of AI in Imaging

Unlike traditional imaging, which relies on qualitative characteristics to identify tumors, including tumor density, enhancement patterns, cell and tissue composition within the tumor, regularity of tumor margins, and anatomical relationships with surrounding tissues, imaging histology converts tumor images into quantitative characteristics for evaluation [20]. AI can use these quantitative imaging properties to understand and effectively identify tumors in images [21].

### 4.1. Application of AI in Kidney Cancer Imaging

The gold standard of treatment for kidney cancer is surgical resection. However, some patients with benign tumors also receive improper surgical excision that leads to unnecessary harm to the patient. In 15% of patients with angiomyolipomas (AML), their nephrectomy is performed improperly [22], while individuals with benign tumors that contain only a small amount of adipose tissue are more likely to be misdiagnosed [23]. Finding renal masses from imaging may not be challenging, but recognizing them is difficult. Meanwhile, contrast-enhanced CT (CE-CT) is a crucial imaging technique for diagnosing renal tumors and reaches a 77–84% accuracy rate for differentiating RCC from benign lesions [24,25]. The ability of AI to distinguish between benign and malignant tissue has been improved in several studies employing texture analysis (TA) methods with CE-CT. Kunapuli et al. trained AI to distinguish between benign and malignant tumors using 100 malignant and 50 benign kidney mass CT images, with a final accuracy of 82% [26]. A more extensive study conducted by Sun et al. comparing manual and ML for differentiating benign from malignant tumors by interpreting CE-CT images revealed that ML could differentiate tumors with an accuracy of 90%. In contrast, experts could only reach 73% accuracy, which indicates that AI would lessen errors [27].

Additionally, Nassiri et al. developed an ML algorithm to discriminate between renal cancer and benign renal tumors using CT images of patients with renal masses and attempted to improve the diagnostic accuracy of the model by incorporating clinical factors. The results showed that the radiology-based prediction model could distinguish between benign and malignant renal masses (AUC = 0.84 (95% CI 0.79–0.9)). Clinical factors did not significantly improve the diagnostic accuracy of the prediction model [28]. Ayman et al. created a renal cancer computer-assisted diagnosis (RC-CAD) system to recognize kidney AML, which contains 3D morphological features, first- and second-order 3D textural features, and time-related renal function indicators. The result showed that RC-CAD reached a sensitivity of (95.29 ± 2.03%) and a specificity of (99.86 ± 0.43%) [29]. On multiphase Magnetic Resonance Imaging (MRI), Ashkan et al. demonstrated an ML to distinguish RCC and oncocytoma with 91% accuracy [30]. Moreover, by interpreting the patients’ CT scans and grading the nuclei, Cui et al. could predict the prognosis of kidney cancer patients [31]. A series of studies have demonstrated the potential for AI to significantly enhance diagnostic accuracy significantly, thereby reducing the incidence of preventable medical errors.

### 4.2. Application of AI in Bladder Cancer Imaging

Based on whether the tumor tissue infiltrates the muscular layer of the bladder, bladder cancer can be divided into NMIBC and MIBC [32]. Correct staging is necessary for treating and predicting the prognosis of bladder cancer, which is usually determined by imaging [33]. Zhang et al. tried to apply AI to forecast the muscle invasiveness of NMIBC. The resulting DL model beat expert radiologists and showed strong prediction power after retrospectively learning CT images of 441 bladder cancer patients (AUC: 0.861 in the internal validation cohort, 0.791 in the external validation cohort) [34]. Yang et al. collected 1200 CT images of bladder cancer patients for DL and constructed eight DL-CNN models. Final results showed that all DL-CNN models distinguished between NMIBC and MIBC well. The VGG16 model had the largest AUROC of 0.997 among the eight algorithms, with a sensitivity and specificity of 0.889 and 0.989 [35]. Moreover, Chen et al. conducted a similar study by retrospectively collecting CT images of 43 patients with MIBC and 130 patients with NMIBC for ML. The results showed that the final model was accurate in determining whether the tumor had infiltrated the muscular layer (AUC: 0.973 (95% CI 0.949–0.999)) [36].

Cystoscopy is one of the most important diagnostic methods for bladder cancer. However, as a subjective test, the doctor’s competence and experience frequently impact the outcomes [37]. According to previous studies, 10–20% of lesions are missed during a cystoscopy [38]. Researchers implemented AI models to increase the examination’s accuracy and avoid the doctor’s subjective effect.

A Googlenet-based CNN model was trained and verified by Atsushi et al. using 2104 pictures of cystoscopy, and the final model’s sensitivity and specificity reached 89.7% and 94.0%, respectively [39]. Wu et al. created a cystoscopic AI using pyramidal scene resolution networks and migration learning through a multi-center study diagnostic system (CAIDS), which included 69,204 consecutive photos from 10,729 patients at six hospitals. The comparison between the AI and urologists revealed that AI showed superior accuracy and sensitivity and was also quicker [40]. Moreover, Zheng et al. conducted a study that is comparable to this one. A ResNeXt-101-32 8d-FPN-based CNN model was used in the study to analyze 10,991 cystoscopy images of probable bladder cancer. The final findings revealed that the diagnostic sensitivity, specificity, and accuracy were 95.0%, 93.7%, and 94.1%, respectively [41]. These studies suggest that AI may help interpret cystoscopy results to lessen the subjective influence of the examiner on the outcomes.

### 4.3. Application of AI in Prostate Cancer Imaging

MRI is one of the most crucial imaging systems for diagnosing and treating prostate cancer. It is frequently used to assist in the diagnosis of prostate cancer and targeted prostate biopsies. Maris et al. reported clinical trials of the prostate puncture robot PROST, which processes images and supports fusion with MRI for targeted biopsies through techniques such as deep learning. This is of great significance in reducing the learning curve for the physician and reducing puncture injuries. However, the doctor’s ability impacts how the MRI is interpreted, and small tumors are frequently disregarded [42]. Winkel et al. compared the accuracy of MRI images of prostate cancer diagnosed by professional or lay physicians with and without DL-CAD, a deep learning-based computer-aided diagnosis (DL-CAD) system. The accuracy increased from 84% to 88% with the help of the AI system, and the agreement between the two diagnoses made by the readers (measured by Fleis K) increased from 0.22 to 0.36 [43]. 

Another study by Labus et al. also evaluated the accuracy of prostate MRI readings made using the DL-CAD system by experts and less experienced doctors. The findings revealed that with AI’s assistance, inexperienced doctors’ diagnostic accuracy was on par with that of experts (AUC = 0.80 vs. AUC = 0.84) [44]. In addition, Yu et al. created PI-RADS.AI, a new AI system for human–computer interaction, to aid in deciphering prostate MRI. In MRI imaging of prostate cancer, PI-RADS identified 87.2% (628/720) of the targeted lesions [45]. A review of the use of AI in prostate cancer MRI interpretation by Turkbey et al. in 2022 showed that the accuracy of AI interpretation in MRI was convincing. However, the clinical benefits need to be followed up with a series of clinical trials that are still required [46].

Due to the low incidence, there needs to be more research on AI in the imaging of the remaining urological cancers.

## 5. AI in Clinical Treatment

Currently, the main treatment methods for urological cancers are radiotherapy, drug therapy, and surgical resection. According to the features of the tumor, each patient should receive a customized course of treatment. Doctors, however, find it challenging to compile this complex information. AI can quickly summarize data from diverse perspectives and assist with treatments. 

### 5.1. AI in Clinical Treatment of Kidney Cancer

Surgical resection is essential for kidney cancer. AR superimposed with preoperative 3D imaging into the surgical field can provide additional information for the surgeon when performing renal surgery. However, it may increase the risk of surgery if superimposed on surgical instruments. Backer et al. improved the AR application technique by using DL to accurately identify surgical instruments in the surgical field. Subsequently, eight surgical cases were performed with satisfactory results [47]. Furthermore, Han et al. developed multiple models using ML for 4104 kidney cancer patients who underwent nephrectomy to predict acute renal injury after the operation. The findings demonstrated that ML outperformed conventional logistic regression (LR) models in terms of predictive power, which means doctors can focus on patients with a higher likelihood of renal injury [48]. Moreover, Amparore et al. developed a software called “IGNITE” (indocyanine GreeN automatIc augmenTed rEality) to perform intraoperative image recognition and enable fully automated hyper-accuracy 3D (HA3D) model overlay. The automatic HA3D overlay was achieved in all ten AR-RAPNs performed [49].

Moreover, predicting the response to immunotherapy is also essential to kidney cancer. Rallis et al. conducted a study to train AI models to predict the response of immunotherapy and targeted therapy with CT image features of RCC [50]. Chen et al. developed and validated the F-box gene family (FBG)-associated novel phenotypes of CCRCC by DL to robustly stratify patients’ survival and immunotherapy response [51]. Various research may allow more people to benefit from immunotherapy.

### 5.2. AI in Clinical Treatment of Bladder Cancer

The standard of treatment for MIBC is radical resection following neoadjuvant chemotherapy. However, unexpected response to chemotherapy is one of the factors preventing neoadjuvant chemotherapy for MIBC patients. Patients with chemotherapy resistance run the risk of tumor progression or potentially losing the chance of surgery [52]. Moreover, no reliable models have been demonstrated to predict treatment response [53] accurately. Cha et al. constructed three different AI models to assess chemotherapy response based on CT images before and after treatment. The final results showed that the AUC of the three models ranged from 0.69–0.77, which was not optimal. Nevertheless, it showed that AI could predict chemotherapy response [54]. In addition, another study conducted by Wu et al. used transfer learning to indicate bladder cancer chemotherapy response by freezing different layers of an existing DL-CNN model. The retrained model’s best AUC was 0.86, superior to the baseline model [55]. 

Neoadjuvant immunotherapy has been recommended for patients with MIBC. Furthermore, Jiang et al. constructed an ML framework to predict the efficacy of immunotherapy for bladder cancer by analyzing immunotherapy biomarkers [56]. These studies show that AI’s application to drug efficacy prediction is entirely feasible.

### 5.3. AI in Clinical Treatment of Prostate Cancer

Radiotherapy is one of the essential treatments for advanced prostate cancers. The contour or boundaries of the radiation object must be carefully planned to destroy tumor tissues while minimizing harm to normal tissues effectively. However, a radiology plan takes a long time to develop, and plans developed by several doctors or the same doctor multiple times often contain significant errors [57]. For radiation region delineation, combining AI with imaging can increase precision and lower mistakes. 

Almeida et al. assessed 28 pertinent studies, of which 19 were analyzed using prostate cancer MRI pictures and 9 using prostate cancer CT images. The results demonstrated the viability and superiority of AI radiation area definition over manual delineation [58]. A prospective trial by McIntosh et al. applied AI to the treatment of prostate cancer, in contrast to most prior AI investigations, which were restricted to simulations or retrospective studies. The study looked into whether a radiotherapy treatment plan created by AI might be used in practice. The result revealed that 72% of treatment plans were chosen for AI-developed plans, 89% of AI-specified treatment plans were deemed clinically appropriate, and the use of AI-developed plans decreased the median time needed for the complete planning process by 60.1% (118 to 47 h) [59]. 

Moreover, a prediction model was created by Auffenberg et al. utilizing ML from 7543 prior prostate cancer treatments. The model may aid in making the most effective treatment plan for patients, and the accuracy of the scheme’s AUC was 0.81. It is important to note that patients can access this model because it has been released online [60].

We still have not found any studies on other urological cancers in the field of clinical treatment.

## 6. AI in the Prediction of Outcome

Prognosis has always been the most critical issue for cancer patients. The prognosis of patients with different tumor stages varies considerably, and even among patients with the same stage, individual differences may lead to very different prognoses [61]. Preoperative prediction of postoperative life expectancy using clinical variables significantly impacts patient surgical strategy. Postoperative prediction of patient prognosis based on surgical outcome, pathological factors, and relevant clinical variables is essential for postoperative treatment planning. AI is more accurate than traditional statistical techniques in predicting patient prognosis [62]. The study conducted by Okyaz et al. included 2 million patients diagnosed with urological cancers in the SEER database. A time series of cancer-specific survival estimates was facilitated by ML of pre- and post-diagnostic features of cancer. The model had good predictive power with a C-index of 0.800 (95% CI: 0.795–0.805) in the validation set [63]. 

### 6.1. AI in Predicting Kidney Cancer Outcomes

Kidney cancer metastasis is a critical determinant of patient survival. Ji et al. developed an ML model (KCBM) utilizing the SEER database of 71,414 kidney cancer patients to evaluate bone metastasis and long-term survival in this population. The KCBM achieved an AUC of 0.8269 (95% CI: 0.8083–0.8425) [64]. Another study conducted by Feng et al. also utilized the SEER database and analyzed data from 39,016 patients to develop six ML models for predicting lymphatic metastasis in kidney cancer. The XGB model demonstrated the best performance with an AUC of 0.916 [65]. 

Patient life expectancy is essential for treatment planning. Mostafa et al. combined imaging and clinical information to train ML models for predicting kidney cancer patients’ 5-year overall survival (OS). The XGBoost model achieved the best performance with AUC, accuracy, sensitivity, and specificity ranging between 0.95–0.98, 0.93–0.98, and 0.93–0.96 [66], respectively. Moreover, survival can also be predicted from a molecular perspective. Zhu et al. utilized an AI to construct a universal molecular prognostic score (mPS) consisting of 21 genes for predicting the prognosis of patients with renal cell carcinoma [67].

### 6.2. AI in Predicting Bladder Cancer Outcomes

Approximately 50% of patients with NMIBC develop MIBC without treatment, and the recurrence rate after treatment is about 70–80% [68]. Existing systems for predicting recurrences, such as the European Organization for Research and Treatment of Cancer (EORTC) and the Spanish Urological Club for Oncological Treatment (CUETO), focus on clinical features such as tumor grading and staging but do not incorporate other information such as imaging and molecular markers. A retrospective analysis including 4689 patients showed that both tools were poor at differentiating between disease recurrence and progression (0.597 and 0.662 for EORTC and 0.523 and 0.616 for CUETO models, respectively) [69]. Jobczyk et al. performed deep learning using clinical variables from the CUETO and EORTC scales, and the final calibrated model showed higher accuracy than the traditional scale. The model had a c-index of 0.650 (95% CI 0.649–0.650) for RFS and 0.878 (95% CI 0.873–0.874) for PFS in the training group [70]. The NMIBC recurrence prediction model trained by Lucas et al. using DL combined with clinical variables as well as pathological images had higher accuracy and specificity than the COX regression model using clinical data alone (0.89 vs. 0.67) [71].

For the 5-year OS in 161,227 bladder cancer patients, Hriday et al. created an artificial neural network (ANN) model and a traditional multivariate Cox proportional prediction model (CPH). Comparing the two models revealed that ANN had an AUC of 0.81, whereas CPH only had 0.70 [72]. Xu et al. used 76 combinations of 10 ML algorithms and small gene sets in the same bladder cancer patient dataset to explore which ML model performs best in predicting survival. The AIGs model with the best performance was finally selected. Patients with high AIGS had a worse prognosis than those with low AIGS and a correspondingly increased risk of disease progression [73]. Similar to Xu’s study, Wang et al. used seven ML algorithms to develop a prediction model for 5-year OS in bladder cancer patients to select the best ML method. Regularized ELM (RELM) was the fastest method among these seven methods, and it had the highest prediction accuracy (80%) [74].

### 6.3. AI in Predicting Prostate Cancer Outcomes

Statistics show that the prostate cancer postoperative biochemical recurrence rate (BCR) might reach 27% [75]. Early intervention can be achieved through BCR prediction; however, conventional models only consider clinical information while ignoring genetic and imaging data [76]. A model for BCR prediction (DRS-BCR) was created by Ye et al. using DL and the MRI data of 485 patients. The DRS-BCR had a higher accuracy than the conventional prediction model [77]. Wei et al. constructed a DL-based BCR prediction model using genomics. 

The study by Lee et al. included data from 171,942 men with prostate cancer in the SEER database to develop an ML model to predict survival. The ML model’s (C-index 0.829, 95% CI 0.820–0.838) predictive power was marginally outperformed by the typical multivariate model’s (C-index 0.820, 0.811–0.829) [78].

### 6.4. AI in Predicting Other Cancer Outcomes

Lymph node metastasis in penile cancer is closely related to treatment planning. Ding et al. trained an ML model to predict lymph node metastasis in penile cancer using data from 1056 penile cancer patients in the SEER database, which provided some assistance in clinical decision-making [79].

## 7. Limitations and Future of AI

**Figure 3:**
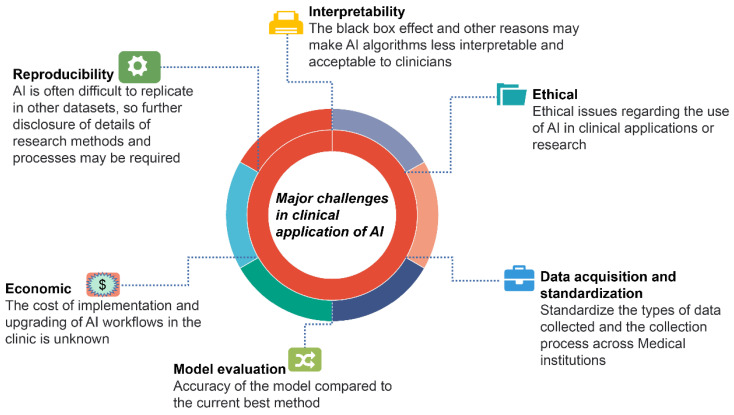
Major challenges in the current application of AI in urological cancers.

Urological cancers have been shown to benefit from AI in terms of diagnosis, prognosis, and treatment. However, there are many limitations in clinical applications (Figure 3). Most AI-related research is based on retrospective data analysis, and prospective studies that validate its use in clinical settings are lacking.

It is challenging to validate and promote the application of external datasets because different research designs, including AI algorithms, observational indicators, and reference data, are inconsistent. Furthermore, some research fails to compare the two models because AI can use large amounts of data to calculate different parameters. In contrast, traditional statistical approaches can only use a limited number of training features. Another issue in the clinical application of AI is the interpretability of the algorithms, which can lead clinicians to doubt and distrust them due to the “black box” effect. The cost of clinically rolling out AI applications and subsequent software updates is currently unknown and may be one of the barriers to AI adoption. What is more, there are currently no guidelines in place to help determine the ethical and legal issues associated with the application of AI.

Fortunately, recent advances in data visualization tools have deepened the visual understanding of algorithmic decisions [80], making it easier to promote optimization techniques and gain widespread clinical acceptance. AI can be advanced and complemented by more accurate and consistent data, thanks to ongoing advances in data collection and standardization of diagnosis and treatment. We believe that as artificial intelligence continues to advance, doctors’ distrust of the technology will diminish as it becomes more widely used in clinical settings. The rationalization of its clinical use will also be supported by improvements in legislation and ethics.

## 8. Conclusions

**Figure 4:**
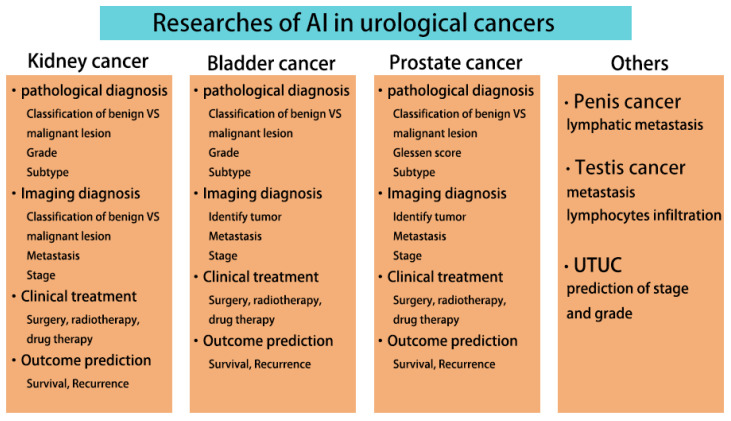
Current AI research in urological cancers.

We summarize the current research areas of AI in urological cancers (Figure 4), where AI has made significant advancements. Current research has focused on the three cancers with greater incidence, kidney, bladder, and prostate cancers, while the rest of the urological cancers are still less studied. Numerous studies have shown that AI is more accurate, faster, and easier to use than traditional methods. Although the clinical application of AI still faces technical and ethical issues, the era of using AI in urological practice is fast approaching, as AI is attracting more and more attention and technological breakthroughs are being made. AI will revolutionize the personalized management of urological cancers throughout the process.

## Data Availability

Not applicable.
